# Drama Therapy Counseling as Mental Health Care of College Students

**DOI:** 10.3390/ijerph16193560

**Published:** 2019-09-23

**Authors:** Wen-Lung Chang, Yu-Shiuan Liu, Cheng-Fu Yang

**Affiliations:** 1 Nanning Normal University; School of Education Science; Nanning 530001, China; jw2008c@hotmail.com; 2 Fujian Normal University; School of Psychology; Fuzhou 350117, China; ziyaliu@hotmail.com; 3 National University of Kaohsiung; Department of Chemical and Materials Engineering, Kaohsiung 811, Taiwan

**Keywords:** Drama therapy, counseling group, college student, mental health

## Abstract

(1) Background: This study aims to apply drama therapy to a counseling group to address the mental health problems of college students in Taiwan due to the increasingly serious psychological problems that have happened in recent times. Based on the healing factors in drama therapy, we applied such therapy activities to four counseling groups composed of 12 high-risk students from Taiwan. (2) Methods: “Questionnaire-based assessment, participant self-assessment and participant attitude assessment” methods were used to evaluate the six mental health indicators of the participants in the evaluation of drama therapy’s effect and the groups’ pre-test and post-test (the first group and the last group). The six indicators were self-awareness, self-expression, interpersonal and communication skills, self-cognitive reconstruction ability, social role ability, and decision-making ability. Data were collected and assessed for the frequencies and percentages of each indicator item. Sets of paired-samples t-tests, independent t-tests, and two-way repeated measures ANOVAs were employed to evaluate the different designs. (3) Results: The results revealed that drama therapy could deliver significantly positive effects for and improve the six mental health indicators of the participants. Males’ self-awareness and decision-making actions were more positively affected than females. (4) Conclusions: The study helps to provide a path of establishing the mental health module of drama therapy in the education sector in Taiwan.

## 1. Introduction

Nowadays, there is increasing demand by college students for mental health counseling due to the increased challenges posed by both the growing number of students with serious psychological problems on campus and the increase in the number of students seeking counseling [1]. According to the Taiwan Ministry of Education, because of the crime news “random homicide of a college student” in Taipei Metro, in which many people were killed or injured [2], Taiwan’s colleges and universities should increase their counseling manpower and strengthen counseling for students to prevent the repetition of regret and potential violent action. In this paper, the implications of mental health problems for college students in Taiwan are discussed, and some strategies for responding to the surge in mental health needs on college campuses are proposed.

### 1.1. Purpose of the Study

Strengthening mental health care for college students through counseling is the primary task of colleges and universities in Taiwan. For students with high-risk mental health, i.e., “major trauma experience and poor life adaptation,” schools should formulate individual counseling and group programs. If further behavioral deviation and major violations occur, they should even strengthen counseling in combination with professionals [2]. In view of increasingly serious mental health problems, researchers have proposed the application of a drama therapy counseling group for college students who have mental health problems in Taiwan.

### 1.2. Literature Review

Drama is a process of role-playing. For human beings as a whole, drama is inherent in the process of simulation and learning, which extends from life [3]. By using these innate instincts and developments and by strengthening the function of learning in education, learners can apply their roles and skills to life, understand their own and other people’s emotions, learn to self-understand and understand other people’s situation, and then have a benign interaction with society [3,4,5,6].

Drama therapy is a term that combines the concepts of drama and therapy [6,7,8]. Literally, it represents a form of drama as a form of therapy. “Drama” refers to a work (script) of “poetry” or “prose” for performers to perform on stage and to simulate the process of life, characters, or narration of a story through the “action” and “interpretation” of performers [6,7,8,9,10,11,12].

Drama therapy differs greatly from traditional counseling methods in that it takes “language” as the main assistant tool. Drama therapy is a combination of professional basis, psychology, sociology and the pedagogy of the “action method” of “drama/theatre” as a tool of psychotherapy and counseling. The process of drama therapy is to overcome the painful limitations of fantasy and daydreaming, bring them to the realm of art, and give them “action performances” to help manage basic conflicts [6,7,8,9,10,11,12].

The “action and performance” process of drama therapy, whether through “theatre games,” “role-playing,” “storytelling” or “creative and expressive artistic activities,” aims at promoting physical and mental harmony and seeking a “mediocre way” in the mind, and these experiences can help individuals living in extreme situations to find out the meaning of life and rebuild their balance so as to acquire ability and try to play a new role; the increase of the role database in the treatment process can also help prepare for the conflicts between roles and situations in future life [6,7,8,9,10,11,12]. 

The six-key model is a way of organizing material in drama therapeutic terms. The model provides an integrative picture of drama therapy processes and furnishes us with six parameters for assessment. The six-key model is shown below [7].

(1) An ability to transport oneself to and from ordinary reality.
(2) A particular quality.
(3) Roles and characters.
(4) Patterns: Plot, themes, and conflicts.
(5) A response to it.
(6) A subtext.

The model serves several purposes [7]:

(1) It allows drama therapists to survey the process.
(2) It organizes the material in a systematic way around six core points.
(3) It reveals the charged parameters.
(4) It helps the drama therapist to choose specific interventions or models, which could more effectively advance the therapeutic process.
(5) It furthers drama therapeutic thinking by conceiving the picture in drama therapy terms.

### 1.3. Significance of the Study

Drama therapy has six major healing indicators for high-risk trial subjects such as the self-awareness ability, self-expression ability, interpersonal and communication skills, self-cognition reconstruction ability, social role ability and decision-making ability of the subjects [3,4,5]. It is applied not only in medical science but also in communities, education fields and other special fields. Based on the healing factors of drama therapy, researchers are encouraged to think about how to apply drama therapy activities to the counseling groups of high-risk students to develop and counsel the psychological potential and sustainability of the college students. The group objectives include the above mentioned six indicators of high-risk students.

### 1.4. Research Questions

The research questions focus on how to apply drama therapy to develop the mental health of high-risk college students in Taiwan, how to evaluate the changes of the six indicators before and after the implementation of the counseling group, and to evaluate the impact of gender differences among participants.

## 2. Materials and Methods

### 2.1. Participants

The object of this drama therapy activity was a participant-centered design. The premise was to understand the correlation between the effect of drama therapy and the age, experience, needs, interests, background, and other basic information of the participants. The participants included 12 students from a college counseling center, with a sex ratio of 7 males to 5 females. Participants background notes: 1. New members: Those who had not yet participated in any counseling center activities; 2. Voluntary participation: Those who were interested in drama therapy counseling groups; 3. Participation: Those who had better participation and cooperation efficiency in drama therapy counseling groups; and 4. High-risk members: Those who had a major traumatic experience, poor life adjustment, behavioral deviation, and major violations. The backgrounds of the participants were provided by the counseling center, and the details and grouping are shown in Table 1.

#### Limitation of the Study

However, this research was not an experimental study. The limitation of the study was its small sample size—12 students from a college counseling center, with a sex ratio of 7 males to 5 females.

### 2.2. Research Design

There were two parts of the design of the study. One was that researchers revised the concept of group therapy according to the theory and technique of drama therapy [6,7,8,9,10,11,12,13,14,15], the concepts of group and arts therapy [16,17,18,19,20,21], and the theory and practice of group psychotherapy. The other part was designing the strategies and implementation methods of the drama therapy counseling group.

#### 2.2.1. Seven Main Elements in the Design of Drama Therapy Counseling Group

1. Relevant resources: This included human resources (such as the leader’s professional skills and experience, and team, organization), material resources (such as equipment and venues) and financial resources (such as activity fees). In addition to providing assistance from the drama therapy counseling group, resources also formed possible constraints.
2. The goals of the drama therapy counseling group: The goal of the drama therapy counseling group was designed by the student counseling center and the researcher gate according to the participants. Group objectives included six indicators of high-risk students’ self-awareness, self-expression, interpersonal and communication skills, self-cognition reconstruction ability, social role ability, and decision-making ability.
3. The theme and content of the drama therapy counseling group: These considered the development stage of the participants and focused on the important issues of personal or life experience, family and social issues.
4. The processes and activities of the drama therapy counseling group: Groups conducted intensive and closed growth counseling groups. The drama therapy group activities were used as the main axis of each unit. The introduction of the theme before group activities and the processes of group warm-ups mainly consisted of the group activities of the drama therapy counseling. In the stage of activities, the mode of “guidance, practice, group interaction, discussion and sharing” was adopted.
5. Time: This could be divided into the arrangement time of the theme, the duration of the activity, the implementation time of the period, and so on. In addition to meeting the needs of the participants, it also cooperated with the college calendar. It was planned to adopt small group counseling mode, which was divided into two stages and ten steps for a total of six months. The 1^st^–4^th^ time adopted intensive battalion courses to build group motivation, and the 5^th^–10th time took place once a month.
6. Leader: The leader was not only the designer of the group and the implementer of the content but also the guide. In the process of the group, the theory, goals and implementation situation of the application of the drama therapy counseling group were reviewed at any time by the action method. Researchers worked with college counselors in curriculum planning and design. Researchers acted as group leaders. The first researcher in the research team was the main leader, the second researcher was the co-leader, and other researchers worked on group evaluation and data integration.

#### 2.2.2. The Implementation Methods of Drama Therapy Student Counseling Group

There were five methods in the drama therapy counseling group.

1. Sociometry: This was one of the techniques to measure interpersonal relationships as well as a tool to measure interpersonal attraction or rejection in a group in order to determine the degree of acceptance of an individual in a group, to discover existing relationships among individuals, and to expose the structure of the group itself [22].
2. Theatre games: These helped in building trust, expression, tacit understanding and group identification [6,7,8,9,10,11,12]. By relaxing the body, opening up the rhythmic experience of the body, releasing body energy in rhythm, the games improved body language and group motivation, stimulating the sensory acuity of various bodies and developing the expressive potential of the body.
3. Mime: This consisted of regarding the body as a clay sculpture, because drama sculpture has infinite possibilities of dramatic moment and expression [23,24]. Using mime could build up important scenes like dreams, life, family, and friends.
4. Mask projection techniques: The use of “mask” as a visual medium in the process of production and performance was used to attempt to help participants reflect their own personality, social competence, and other aspects, leading to the inquiry of specific roles or situations. Role characters were created and the internal contradictions of different types of characters in the process of playing other people’s roles were experienced so as to carry out interpersonal psychological interactions [6].
5. Playback theatre: This drew material from life stories as theatre materials. Using “fluid sculpture,” the most basic deductive form of playback theatre, allowed for a strong and fast rhythm that was especially suitable for capturing simple feelings. Actors took turns to appear one after another on join the stage merging into a “man-made statue” to make up the feeling of the storyteller [25].

### 2.3. Procedures

The methods and main elements in the design of the drama therapy counseling group the course plan are shown in Table 2.

The following is the activity procedure of each drama therapy student counseling group:

1. Pre-Event Meeting (30–40 min)
  (1) Discuss (the group participants/objectives/strategies) and adjust the reporting process according to the response of the last participants.
  (2) Report process and matters needing attention.
  (3) Preparation (warm-up of leaders and group workers)
2. Implementing Drama Therapy Student Counseling Group (3 hours)
  (1) Warm up (about 30–50 min), such as drama games and social atoms.
  (2) Main activities (about 60–90 min): Personal-group relationships, such as trust and tacit understanding.
  (3) Share for ending (about 30 min): Each meeting: A. Group circle; B. Leader reminds the group of the principle of confidentiality and respect; C. Growth and feeling of participation today; D. Best wishes; E. Looking forward to the next meeting.
3. Post-Event Meeting (45 min)
  (1) Discuss record, evaluation or discovery of activities.
  (2) Revise the next curriculum activity proposal according to participants and staff.
  (3) Develop strategies.

#### 2.3.1. Evaluation

Quantitative data collection and analysis are presented as the results of the group evaluation in this study.

#### 2.3.2. Data Collection

A “questionnaire-based assessment, participant self-assessment and participant attitude assessment” designed by drama therapy experts and scholars [3,4,5,6,7,8,9,10,11] in the evaluation of drama therapy, the group’s pre-test and post-test (the first group and the last group) methods were used to evaluate the change of the six indicators of participants, including A. self-awareness, B. self-expression, C. interpersonal and communication skills, D. self-cognitive reconstruction ability, E. social role ability, and F. decision-making ability, each with a total score of 100 points to represent the change of the process.

#### 2.3.3. Data Analysis

Data were assessed for the frequencies and percentages on each item. Sets of paired-samples t-tests, independent t-tests, and a two-way ANOVA analysis was employed to evaluate the different designs.

## 3. Results

The main reason why researchers choose drama therapy for student counseling groups is that it provides an explorable flexible space. Under the mode of group operation, drama therapy can develop the life and psychological potential of counseling students, with group objectives including the six indicators of high-risk students as defined above, i.e., self-awareness, self-expression, interpersonal and communication skills, self-cognition reconstruction ability, social role ability, and decision-making ability.

### Results of Assessments

The following is an analysis of the results of the implementation of the drama therapy student counseling group.

In this study, the results of the paired t-tests are shown in Table 3. The overall evaluation shows that drama therapy had a significant effect on the participants, t = −34.48, *p* < 0.01. Several significant effects for the dimensions of evaluation were found, with-subjects paired-sample t-tests revealed that drama therapy significantly increased the participants’ scores for self-awareness, t = −13.54, *p* < 0.01; self-expression, t = −11.31, *p* < 0.01; interpersonal interaction and communication skills; t = −12.21, *p* < 0.01; self-recognition ability; t = −34.12, *p* < 0.01, social role ability; t = −20.90, *p* < 0.01; and decision-making ability, t = −12.90, *p* < 0.01. Thus, the result revealed that drama therapy could deliver significantly positive effects and improve the self-awareness, self-expression, interpersonal interaction and communication skills, self-recognition ability, social role ability, and decision-making ability of participants.

Two-way repeated measures ANOVA results showed a significant two-way interaction between gender and the six dimensions of evaluation, whereby drama therapy more positively affected female participants’ perception of self-awareness (F = 9.10, *p* < 0.05, and decision-making ability, F = 5.56, *p* < 0.05) than males. Males with a differentiation of self-awareness (Mpre-post = −11.43) showed a stronger negative range score than females with a differentiation of self-awareness (Mpre-post = −8.00). Males with a differentiation of decision action (Mpre-post = −8.28) showed a stronger negative range score than females with a differentiation of decision action (Mpre-post = −6.00). There was no significant interaction between evaluations of self-expression, interpersonal interaction and communication skills, self-recognition ability, and social role ability and gender (Table 4). Thus, the results revealed an observable interaction between the self-awareness, decision-making ability, and gender of participants. Males had more positive effects on the self-awareness and decision action than females after participating in the drama therapy group.

As predicted in the study, two significant two-way interactions were found between the four symptoms and the six dimensions of evaluation. Participants with the symptom of behavioral bias reported a stronger negative range score in the differentiation of interpersonal interaction and communication skill than those with the symptom of maladaptation (F(3,8) = 4.07, *p* < 0.05, Mpre-post/behavioral bias-maladaptation = 10.00). Participants with the symptoms of behavioral bias and trauma reported a stronger negative range score in the differentiation of interpersonal interaction and communication skill than those with the symptom of violation (F(3,8) = 8.00, *p* < 0.05, Mpre-post/ behavioral bias-violation = 8.00, Mpre-post/trauma-violation = 10.00). There was also no significant interaction between evaluations of self-awareness, self-express, self-recognition ability, and social role ability with the symptoms of violation, behavioral bias, maladaptation, and trauma (Table 5). Thus, the results revealed that the interaction between symptoms and drama therapy improved participants’ symptoms. Participants with a behavioral bias had stronger depression in the interpersonal interaction and communication skill than those with maladaptation. Participants with the symptoms of behavioral bias and trauma showed a quicker decision action than those with the symptom of violation.

## 4. Discussion

In this study, researchers applied drama therapy methods to develop the mental health of 12 members of high-risk college students in Taiwan, with a sex ratio of seven males to five females. The researchers evaluated the changes of the six indicators before and after the implementation of the counseling group and the impact of gender differences among the participants.

The drama therapy group members’ scores in the pre-test and post-test changes of the questionnaire, self-assessment, and participation attitude were significantly improved.

Firstly, the biggest change in the chart was the “self-perception” part of the members. It could be seen that the drama therapy groups had the greatest effect on enhancing participants’ self-awareness ability. The main reason for this is that the drama therapy groups focused on how to help members to “materialize” their hidden feelings by means of action so that their inherent feelings, which are not easily perceived, could be seen and discovered. Next, in the “self-expression ability” section, it was also found that the drama therapy groups improved the members’ self-expression ability. The effect was excellent. To those who are not good at using spoken language to communicate with others, it increases many chances of communicating freely, such as verbal and body language. In addition, by telling life stories and roles, the drama therapy groups also helped members who had trouble expressing their feelings to share their inner feelings with others. Finally, all six indicators of the group’s goal of participant counseling showed obvious improvement.

## 5. Conclusions

The application of drama therapy in the education field can strengthen the function of learning in education. The learners can use the skills of roles in life, understand their own and other people’s emotions, and then have a positive interaction with society. Because of the “random killing of college students” in the Taipei Metro and the increasingly serious problems faced by students from high-risk families in Taiwan, researchers have applied drama therapy to the counseling group of Taiwanese’ high-risk college students’ mental health.

Suggestions for future researchers: Drama therapy evolved mostly out of fieldwork. Thus, like a craft that incorporates professionals from various disciplines, researchers have tended to lean on their own backgrounds when choosing their methods. The researchers should have drama therapy and group counseling training and supervise to ensure participants’ well. Learning to deal with participants’ complicated backgrounds is also important. Drama therapy counseling groups require a lot of personal interactions including physical and verbal communication. It is challenging to build up group security and trust, so effective theatre games can build good relationships and lay good foundations for research. Each group and individual has their own characteristics, so researchers should be sure to customize their program accordingly.

At present, there are not many studies on the application of drama therapy in the school counseling field in China. The study provides a path to establish a mental health module of drama therapy in the education field of Taiwan.

## Figures and Tables

**Table 1:** Background and grouping of the drama therapy student participants.

Participants’ Code	Gender	Age	Level	New Members	Voluntary Participation	High-Risk Situation
A	Male	20	Junior	Yes	Yes	Major violations
B	Male	18	Freshman	Yes	Yes	
C	Male	19	Sophomore	Yes	Yes	
D	Male	20	Junior	Yes	Yes	Behavioral deviation
E	Female	19	Freshman	Yes	Yes	
F	Male	21	Senior	Yes	Yes	
G	Male	20	Junior	Yes	Yes	Poor life adjustment
H	Female	18	Freshman	Yes	Yes	
I	Female	20	Sophomore	Yes	Yes	
J	Male	19	Freshman	Yes	Yes	Major traumatic experience
K	Female	21	Junior	Yes	Yes	
L	Female	20	Sophomore	Yes	Yes	

**Table 2:** Drama therapy student counseling group course plan.

Course Time	Theme	Participants
1st	Warm up: Sociometry (establish a group). Building a sense of trust. Sociogram: Drawing my life.	1. Leader, 2. Co-leader, 3. Students.
2nd	Warm up: Theatre games. Mime: Sculpture of my friend/dream/family. Share for ending.	1. Leader, 2. Co-leader, 3. Students.
3rd	Warm up: Theatre games. Group statues: Family chorus. Mask making.	1. Leader, 2. Co-leader, 3. Students.
4th	Warm up: Theatre games. Mask practice: My home. Share for ending.	1. Leader, 2. Co-leader, 3. Students.
5th	Theatre games (energy warming up). Storytelling and fluid sculpture. Share for ending.	1. Leader, 2. Co-leader, 3. Students.
6th	Warm up: Theatre games. Playback theatre. Share for ending.	1. Leader, 2. Co-leader, 3. Students.
7th	Warm up: Theatre games. Role-playing. Share for ending.	1. Leader, 2. Co-leader, 3. Students.
8th	Warm up: Theatre games. Storytelling theatre: Life story presentation practice. Share for ending.	1. Leader, 2. Co-leader, 3. Students.
9th	Warm up: Theatre games. Life story presentation. Share for ending.	1. Leader, 2. Co-leader, 3. Students.
10th	Warm up: Theatre games. Blessing ceremony. Group ending.	1. Leader, 2. Co-leader, 3. Students.

**Table 3:** Paired t-test: The dimensions of evaluation.

Dimensions	Mean before Therapy	Mean after Therapy	t Value	*p* Value
Overall	68.21	76.19	−34.48 **	0.00
Self-awareness	65.67	75.67	−13.54 **	0.00
Self-expression	70.17	79.42	−11.31 **	0.00
Interpersonal interaction and communication skills	67.08	73.25	−12.21 **	0.00
Self-recognition ability	66.17	73.83	−34.12 **	0.00
Social role ability	71.67	79.17	−20.90 **	0.00
Decision-making ability	68.50	75.83	−12.90 **	0.00

Note. ** sig < 0.01.

**Table 4:** Repeated measures ANOVA: The dimensions of evaluations and gender.

Dimensions	df	Gender	Before Drama Therapy	After Drama Therapy	F	*p* Value
Self-awareness and Gender	1	Male	63.43	74.86		
		Female	68.8	76.8		
					9.10 *	0.1
Self-expression and Gender	1	Male	59.86	78.14		
		Female	70.6	81.12		
					2.15	0.17
Interpersonal interaction and communication skills and Gender	1	Male	67.43	73.29		
		Female	66.6	73.2		
					0.5	0.5
Self-recognition ability and Gender	1	Male	63.71	71.71		
		Female	69.6	76.8		
					3.89	0.08
Social role ability and Gender	1	Male	70.29	78		
		Female	73.6	80.8		
					0.48	0.51
Decision-making ability and Gender	1	Male	66.29	74.57		
		Female	71.6	77.6		
					5.56 *	0.4

Note. * sig. = 0.05; N _male_ = 7, N _female_ = 5.

**Table 5:** Repeated measures ANOVA: The dimensions of evaluations and symptoms.

Dimensions	df	Symptoms	Before Drama Therapy	After Drama Therapy	F	*p* Value	Compare
Self-awareness and symptoms	3	Violation	62	72.67		0.47	
		Behavior	68	79.33	0.93		
		Maladaptation	65.33	75.33			
		Trauma	67.33	75.33			
Self-expression and symptoms	3	Violation	72	79.67	2.15	0.5	
		Behavior	69.33	80			
		Maladaptation	64.67	76			
		Trauma	70.17	82			
Interpersonal interaction and communication skills and symptoms	3	Violation	70.33	74.67		0.05	
		Behavior	70.33	76.33	0.67		
		Maladaptation	59.33	67.33			B>M
		Trauma	68.33	74.66			
Self-recognition ability and symptoms	3	Violation	64.67	72.67		0.6	
		Behavior	66.67	74	4.07 *		
		Maladaptation	64	71.33			
		Trauma	69.33	77.33			
Social role ability and symptoms	3	Violation	70	78.67		0.02	
		Behavior	74	81.33	5.83		
		Maladaptation	69.33	75.33			
		Trauma	73.33	81.33			
Decision action and symptoms	3	Violation	62	70.67		0.01	
		Behavior	70	78.67	8.00 **		
		Maladaptation	69.33	74			B>V
		Trauma	72.67	80			T>V

Note. * Sig. < 0.05, ** Sig < 0.01; N _Violation_ = 3, N _Behavior_ = 3, N _Maladaptation_ = 3, N _Trauma_ = 3.
